# Electrophysiological and behavioural responses of *Stegobium paniceum* to volatile compounds from Chinese medicinal plant materials

**DOI:** 10.1002/ps.7012

**Published:** 2022-06-15

**Authors:** Yu Cao, Onofrio Marco Pistillo, Yibin Lou, Ilaria D'Isita, Filippo Maggi, Qiqi Hu, Giacinto Salvatore Germinara, Can Li

**Affiliations:** ^1^ Guizhou Provincial Key Laboratory for Rare Animal and Economic Insect of the Mountainous Region, Department of Biology and Engineering of Environment Guiyang University Guiyang People's Republic of China; ^2^ Department of Agriculture, Food, Natural Resources and Engineering University of Foggia Foggia Italy; ^3^ Chemistry Interdisciplinary Project (ChIP), School of Pharmacy University of Camerino Camerino Italy

**Keywords:** *Stegobium paniceum*, EAG, olfactory response, Chinese medicinal plant material, volatile organic compounds, concentration

## Abstract

**BACKGROUND:**

*Stegobium paniceum* (Coleoptera, Anobiidae) is an important pest of stored products causing severe damage to dried Chinese medicinal plant materials (CMPMs). Plant volatiles play an important role in host‐searching of insects. The olfactory responses of *S. paniceum* to the most abundant volatile components of some drugstore attractant CMPMs such as *Panax notoginseng*, *Angelica sinensis*, *Gastrodia elata* and *Peucedanum praeruptorum*, namely falcarinol, 3‐*n*‐butylphthalide, *p*‐cresol and β‐pinene, respectively, were studied by electroantennography (EAG) and behavioural bioassays in six‐ and four‐arm olfactometers.

**RESULTS:**

EAG recordings showed that male and female antennae are able to perceive the test compounds in a wide range of concentrations and in a dose‐dependent manner. Moreover, for each dose of different compounds tested, no significant differences were found between the mean male and female EAG responses. In six‐arm olfactometer bioassays, *S. paniceum* exhibited positive responses to falcarinol, 3‐*n*‐butylphthalide, *p*‐cresol and β‐pinene at doses of 1, 10, 100, 500 and 1000 μg. The most attractive dose was 500 μg for falcarinol, 100 μg for 3‐*n*‐butylphthalide, 500 μg for *p*‐cresol and 1000 μg for β‐pinene. Olfactory preferences of *S. paniceum*, based on comparison of these four compounds at their optimally attractive concentrations in a four‐arm olfactometer, were 3‐*n*‐butylphthalide > *p*‐cresol > falcarinol > β‐pinene.

**CONCLUSION:**

The results indicated that the four volatiles of CMPMs are perceived by the peripheral olfactory system of *S. paniceum* adults and are able to individually elicit a positive chemotaxis in *S. paniceum* adults confirming the role of chemical cues in host‐plant detection and selection of this pest. Further field studies are needed to evaluate the potential of the attractive compounds identified in this study, particularly 3‐*n*‐butylphthalide, to be applied as a novel monitoring and control tool against this storage‐beetle pest. © 2022 The Authors. *Pest Management Science* published by John Wiley & Sons Ltd on behalf of Society of Chemical Industry.

## Graphical abstract

Four compounds identified from the volatiles of Chinese medicinal plant materials **(**CMPMs**)** are perceived by the peripheral olfactory system of *S. paniceum* adults and are able to individually elicit a positive chemotaxis in *S. paniceum* adults confirming the role of chemical cues in host‐plant detection and selection of this pest.

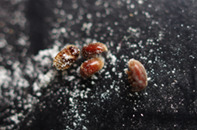

## 1 INTRODUCTION

The drugstore beetle, *Stegobium paniceum* L. (Coleoptera, Anobiidae), is a worldwide pest of stored products. This cosmopolitan insect consumes a wide range of dried plant products, including biological specimens in museum collections.^1^, ^2^, ^3^ *Stegobium. paniceum* adults typically gnaw their way into food storage containers causing direct damage, and then proceed to lay eggs in the stored products. The hatched larvae then infest these stored materials, with the dead beetles and other wastes remaining inside, causing further spoilage and economic loss.^1^, ^2^

Currently, fumigants (e.g. phosphine) are considered to provide the most effective means of protecting and disinfesting stored food and other products.^4^, ^5^ However, the repeated and intensive use of various fumigants has resulted in serious problems, including residues with hazard for human health, pest resistance, pest resurgence and lethal effects on nontarget organisms.^6^ Therefore, the development of effective alternative methods to control pests of stored products is needed. Integrated pest management (IPM) is considered a more sustainable and environmentally friendly control strategy for combatting these pests.^3^, ^5^, ^7^

Behavioural manipulation based on the responses of insects to special environmental factors is now an important method of pest control. Thus, certain repellents and attractants, whose functional components are derived from specific plants, are applied to manipulate the behaviour of insects to protect stored products from pest infestation.^8^, ^9^, ^10^, ^11^ In our previous study, *S. paniceum* adults showed significant preferences for volatiles released from several Chinese medicinal plant materials (CMPMs).^12^ However, specific volatile components of CMPMs that attracted *S. paniceum* were not identified.

Medicinal plants are widely grown in China and make a substantial contribution to the economy. *S. paniceum* is the main pest species responsible for major losses of stored CMPMs in many provinces of China.^1^ Because most of these CMPMs are stored before use in human health protection and disease treatments, it is important to devise methods for the management of *S. paniceum* without risk of contamination of the products. Therefore, there is a keen interest in the development of botanical pesticide alternatives to chemical insecticides for *S. paniceum* control. Indeed, there is a long history of using botanical insecticides to protect stored products from insect pests.^10^, ^13^, ^14^ However, volatile compounds or other plant products also could be developed as attractants or repellents for pest management. In our previous study, volatile blends from various CMPMs, including *Panax notoginseng* (Burkill) F.H.Chen (Apiales: Araliaceae), *Angelica sinensis* (Oliv.) Diels (Apiales: Apiaceae), *Gastrodia elata* Blume (Asparagales: Orchidaceae) and *Peucedanum praeruptorum* Dunn (Apiales: Apiaceae), were strongly attractive to *S. paniceum*, with the most abundant components of these four CMPMs being falcarinol, 3‐*n*‐butylphthalide, *p*‐cresol and β‐pinene, respectively.^12^

In the present study, the olfactory responses of *S. paniceum* to these compounds were investigated by electroactennographic (EAG) tests to assess the sensitivity of male and female *S. paniceum* antennae to the test compounds and by six‐ and four‐arm olfactometer bioassays to evaluate the insects' behavioural response to different concentrations of the same compounds. Such information will help to further elucidate the roles of chemical signals in host selection by *S. paniceum* and provide a basis for further field studies aiming at developing semiochemical‐based strategies for the safe and effective control of this beetle pest.

## 2 METHODS AND MATERIALS

### 2.1 Insect rearing

*Stegobium paniceum* have been reared in the laboratory of the Department of Biology and Engineering of Environment, Guiyang University since 2017 and maintained on jujube (*Ziziphus jujuba* Mill.); a laboratory colony from the same population was established at the Department of Agriculture, Food, Natural Resources and Engineering, University of Foggia. The beetles were reared at 28 ± 1 °C, 60 ± 5% relative humidity, and 8 h:16 h, light:dark photoperiod (photophase 09:00 h and 17:00 h), as reported by Li *et al*.^1^ Secondary infestation by moisture‐sensitive mites was prevented using the method of Steiner *et al*.^15^

### 2.2 Odour stimuli

Falcarinol was purchased from GlpBio (Montclair, NJ, USA), *p*‐cresol and β‐pinene were purchased from Dr. Ehrenstorfer GmbH (Augsburg, Germany), and 3‐*n*‐butylphthalide was purchased from Toronto Research Chemicals (North York, Canada). For each compound, mineral oil (Sigma‐Aldrich, Milan, Italy) solutions to be used as test stimuli for EAG (0.001, 0.01, 0.1, 1, 10 μg μL^−1^) and olfactometer (0.1, 1, 10, 50 and 100 μg μL^−1^) bioassays were prepared. Solutions were stored at −20°C until needed.

### 2.3 Electroantennography (EAG)

The antennal sensitivity of *S. paniceum* males and females to increasing concentrations of the four test compounds was evaluated by EAG using the technique described in our previous studies.^16^, ^17^ The head of a one‐week‐old specimen was excised using a scalpel and seated between two glass capillary (Micro‐glass, Naples, Italy) electrodes filled with Kaissling saline solution^18^ and mounted in stainless steel electrode holders (Syntech Laboratories, Hilversum, the Netherlands). The recording electrode (diameter ~ 100 μm) was put in contact with the dorsal surface of the last antennal segment while the neutral electrode was inserted into the base of the head. AgCl‐coated silver wires were used to maintain the electrical continuity between the antennal preparation and an AC/DC UN‐6 amplifier in DC mode (Syntech Laboratories). The amplifier was connected to a PC equipped with the EAG 2.0 program (Syntech Laboratories).

For each test compound, 10 μL of different mineral oil solutions giving the 0.01, 0.1, 1, 10 and 100 μg doses, was adsorbed onto a filter paper (Whatman No. 1) strip (2 cm^2^) inserted in a Pasteur pipette (15 cm long) which was used as an odour cartridge. Using a disposable syringe, vapour stimuli (3 cm^3^) were puffed for 1 s (0.35 m s^−1^) into a charcoal‐filtered and humidified air flow (500 mL min^−1^) passing over the antenna through a stainless‐steel delivery tube [1 cm inner diameter (i.d.)] whose outlet was positioned ~1 cm from the antenna. Control (10 μL mineral oil) and standard (5 μL of 10 μg μL^−1^ (*Z*)‐3‐hexenol mineral oil solution; Sigma‐Aldrich) stimuli also were applied at the beginning of the experiment and after each group of four test stimuli. The intervals between stimuli were 1 min. Each dose of the four compounds was tested on five different antennae from different males and females.

The maximum amplitude of negative polarity deflection (–mV) elicited by a stimulus was used to measure the EAG responses.^17^ To compensate for solvent and/or mechanosensory artifacts, the absolute amplitude (mV) of the EAG response to each test stimulus was subtracted by the mean EAG response to the two nearest solvent controls.^18^ Moreover, to compensate for the decrease in the antennal responsiveness during the experiment, the resulting EAG amplitude was corrected according to the reduction of the EAG response to the standard stimulus.^19^ Dose–response curves were calculated based on the corrected EAG values.

### 2.4 Six‐arm olfactometer bioassays

The behavioural responses of adult *S. paniceum* to falcarinol, 3‐*n*‐butylphthalide, *p*‐cresol and β‐pinene solutions were evaluated in a six‐arm olfactometer according to the method reported by Cao *et al*.^20^ Each arm was connected to a 25‐mL glass vessel via Teflon® tubing, and each glass vessel contained a test or control stimulus (10 μL). Aliquots (10 μL) of each of the five concentrations (0.1, 1, 10, 50 and 100 μg μL^−1^) of each compound and mineral oil (used as control), adsorbed onto a filter paper disk (1.0 cm diameter), were used as stimuli.

In order to drive the odour source towards the insects, the airflow was set at 200 mL min^−1^. *Stegobium paniceum* unsexed adults, 2–3 days postemergence and starved for 4 h, were introduced into the olfactometer in groups of 180 individuals, using a brush. After 30 min, *S. paniceum* that entered the arms of the olfactometer were counted and considered as either having made a choice for a particular odour source, or were considered as ‘nonresponders’. Bioassays were replicated six times and carried out between 09:00 h and 17:00 h, at room temperature (RT; 25 ± 1 °C). After each replication, the olfactometer was cleaned, dried and the arms were rotated (60°).^12^

### 2.5 Four‐arm olfactometer bioassays

In the six‐arm olfactometer bioassays, falcarinol, 3‐*n*‐butylphthalide, *p*‐cresol and β‐pinene showed the highest attractiveness to *S. paniceum* at concentrations of 50, 10, 50 and 100 μg μL^−1^, respectively. Therefore, the attractant power of the four compounds at their optimal concentrations were compared in a four‐arm olfactometer, using the method of Liu *et al*.^21^ The olfactometer consisted of a central glass chamber (15 cm i.d., 6 cm length) with four arms (1.5 cm i.d., 10 cm length), each connected to a glass tube (1.5 cm i.d., 20 cm length). Each arm was connected via Teflon® tubing to a 25‐mL glass vessel that contained the test or control stimulus (10 μL), and the airflow was set at 200 mL min^−1^. Beetles were introduced into the central part of the olfactometer chamber in groups of 120 individuals. ‘Responders’ and ‘nonresponders’ were determined using the criteria described for the six‐arm olfactometer bioassays. Bioassays were replicated six times and were carried out between 09:00 a.m. and 5:00 p.m, at RT (25 ± 1 °C). After each replication, the olfactometer was cleaned, dried and the arms were rotated (90°). The odour treatments were set as follows:

1. mineral oil, falcarinol, 3‐*n*‐butylphthalide and *p*‐cresol;
2. mineral oil, falcarinol, 3‐*n*‐butylphthalide and β‐pinene;
3. mineral oil, falcarinol, *p*‐cresol and β‐pinene;
4. mineral oil, 3‐*n*‐butylphthalide, *p*‐cresol and β‐pinene.

### 2.6 Statistical analysis

In order to verify antennal activation, the corrected mean EAG response of males and females to the last dilution of each test compound was compared to a ‘0’ value using one‐sample Student's *t*‐test and regarded as ‘activated’ if significant at *P* = 0.05. Saturation level was taken as the lowest dilution at which the mean response was equal to or less than the previous one.^22^ The mean EAG responses of males and females to each stimulus were compared using Student's *t*‐test for independent samples at *P* = 0.05. Male and female EAG responses to each test stimulus were not significantly different; therefore, they were pooled and analyzed together. For each of the 0.1, 1, 10 and 100 μg doses of the four compounds the mean EAG responses of adult *S. paniceum* were submitted to ANOVA followed by Tukey's honestly significant difference (HSD) test (*P* = 0.05) for separation of means. Data were log_10_x‐transformed to satisfy the assumption of normality (Shapiro–Wilk test) and assessed for homogeneity of variances (Levene's test) before ANOVA.

Because there was no difference in response to odour stimuli between *S. paniceum* males and females,^12^ the male and female responses in different behavioural bioassays were pooled and analyzed together. The numbers of insects found in the different arms of the six‐arm and four‐arm olfactometers were subjected to Friedman two‐way ANOVA by ranks and, in the case of significance (*P* < 0.05), the Wilcoxon signed ranks test was used to determine differences among means. All statistical analyses were performed using SPSS 18.0 for Windows (SPSS Inc., Chicago, IL, USA).

## 3 RESULTS

### 3.1 EAG

**Figure 1:**
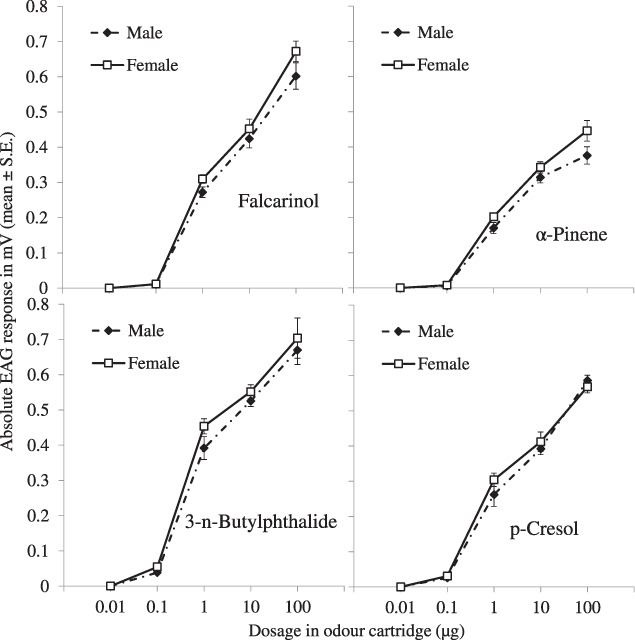
Mean (± SE) EAG dose–response curves of male and female *S. paniceum* antennae to ascending doses of falcarinol, 3‐*n*‐butylphthalide, *p*‐cresol and β‐pinene. For each dose, mean male and female EAG responses were not significant different (Student's *t*‐test for independent samples, *P* = 0.05).

The EAG responses of *S. paniceum* males and females to increasing doses of falcarinol, 3‐*n*‐butylphthalide, β‐pinene and *p*‐cresol are reported in Fig. 1. All compounds elicited measurable EAG responses starting from the 0.1‐μg dose (*P* < 0.05 in all one‐sample Student's *t*‐test). In the dose range tested, typical sigmoid‐shaped dose–responses were elicited by test compounds in both males and females. The mean EAG response to the highest dose was higher than that to the previous dose for all compounds indicating that no saturation of olfactory receptors occurred at the lowest dose. For each dose of the four compounds no significant differences were found between the mean EAG responses of males and females (falcarinol: *t* = 0.447–1.965, df = 8, *P* = 0.086–0.667; 3‐*n*‐butylphthalide: *t* = 1.095–1.843; df = 8, *P* = 0.103–0.305; β‐pinene: *t* = 0.485–1.818; df = 8, *P* = 0.107–0.640; *p*‐cresol: *t* = 0.615–1.175; df = 8, *P* = 0.274–0.556).

**Figure 2:**
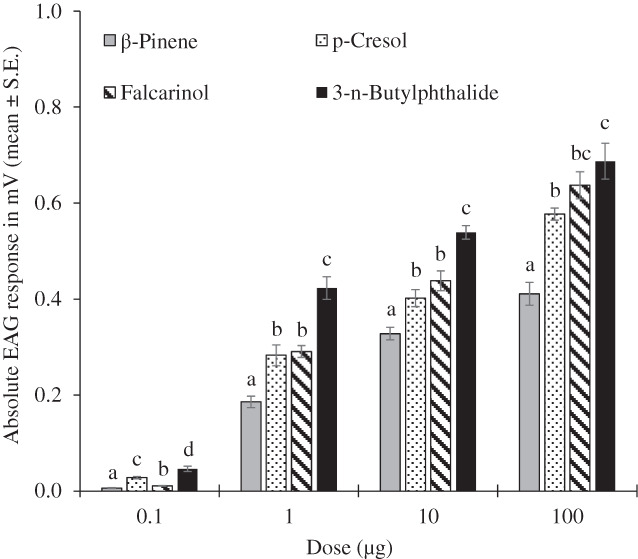
EAG responses of male and female *S. paniceum* antennae to different doses of falcarinol, 3‐*n*‐butylphthalide, *p*‐cresol and β‐pinene. For each dose, different letters indicate significant differences (Tukey's HSD test, *P* < 0.05).

ANOVA revealed significant differences among the mean pooled male and female EAG responses to the four compounds at the 0.1 (*F* = 89.598, df = 3, *P* < 0.001), 1 (*F* = 34.870, df = 3, *P* < 0.001), 10 (*F* = 34.216, df = 3, *P* < 0.001) and 100 μg (*F* = 28.813, df = 3, *P* < 0.001) doses. At 0.1, 1 and 10 μg, the mean EAG responses elicited by 3‐*n*‐butylphthalide were significantly higher than those induced by falcarinol, *p*‐cresol and β‐pinene (*P* < 0.05, Tukey's HSD test) (Fig. 2). At 100 μg, the EAG responses to 3‐*n*‐butylphthalide and falcarinol were statistically similar and significantly higher than those to *p*‐cresol and β‐pinene (*P* < 0.05, Tukey's HSD test).

### 3.2 Six‐arm olfactometer bioassays

**Figure 3:**
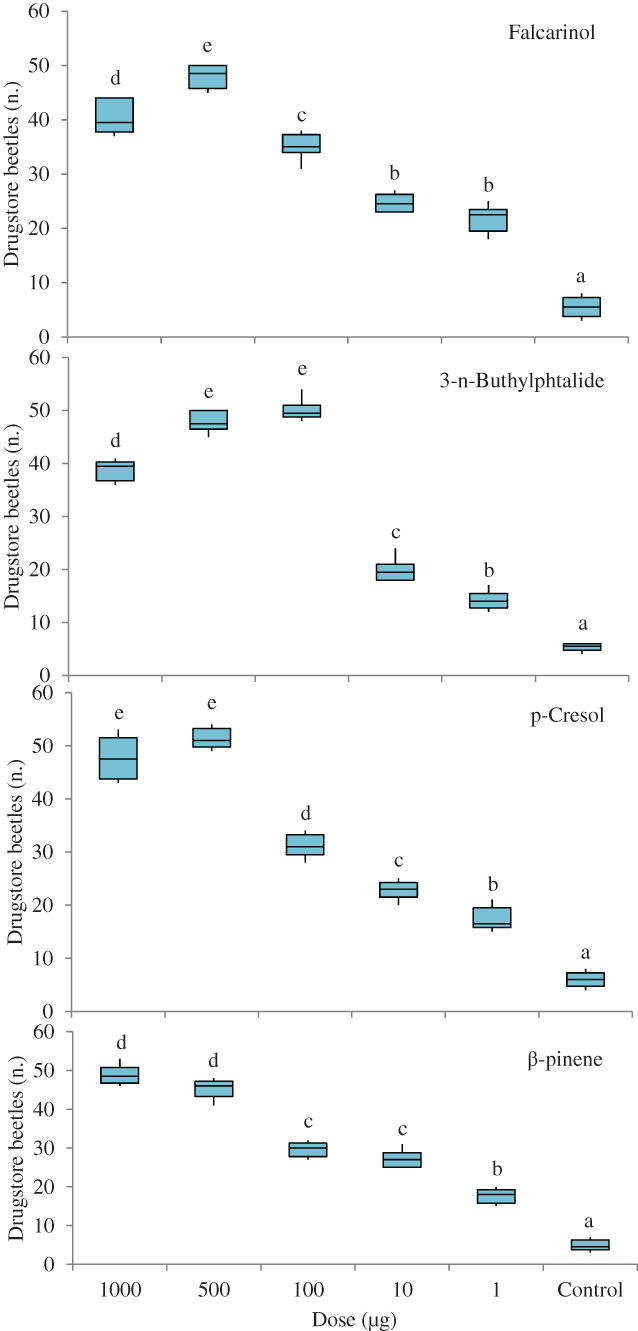
Olfactory responses of *S. paniceum* to different doses of falcarinol, 3‐*n*‐butylphthalide, *p*‐cresol and β‐pinene in a six‐arm olfactometer. The control was mineral oil. Each box plot represents the median and its range of dispersion (lower and upper quartiles and outliers). Above each box plot, different letters indicate significant differences (Wilcoxon test, *P* < 0.05).

In these bioassays, mineral oil controls were significantly less attractive than all doses tested of falcarinol (Friedman test: χ^2^ = 29.498, df = 5, *P* < 0.001; Wilcoxon tests: *P* = 0.027–0.028), 3‐*n*‐butylphthalide (Friedman test: χ^2^ = 29.238, df = 5, *P* < 0.001; Wilcoxon tests: *P* = 0.026–0.027), *p*‐cresol (Friedman test: χ^2^ = 29.238, df = 5, *P* < 0.001; Wilcoxon tests: *P* = 0.027–0.028) and β‐pinene (Friedman test: χ^2^ = 29.048, df = 5, *P* < 0.001; Wilcoxon tests: *P* = 0.026–0.028) (Fig. 3).

There also were significant differences in attractiveness among different doses of each compound. Significantly more insects entered the arms connected to the vessels that contained 500 μg falcarinol (Wilcoxon tests: *P* = 0.024–0.028), 100 μg (Wilcoxon tests: *P* = 0.027) and 500 μg (Wilcoxon tests: *P* = 0.024–0.027) of 3‐*n*‐butylphthalide, 500 μg (Wilcoxon tests: *P* = 0.026–0.027) and 1000 μg (Wilcoxon tests: *P* = 0.027–0.028) of *p*‐cresol, and 500 μg (Wilcoxon tests: *P* = 0.024–0.027) and 1000 μg (Wilcoxon tests: *P* = 0.027) of β‐pinene relative to the arms with the other doses (Fig. 3).

### 3.3 Four‐arm olfactometer bioassays

**Figure 4:**
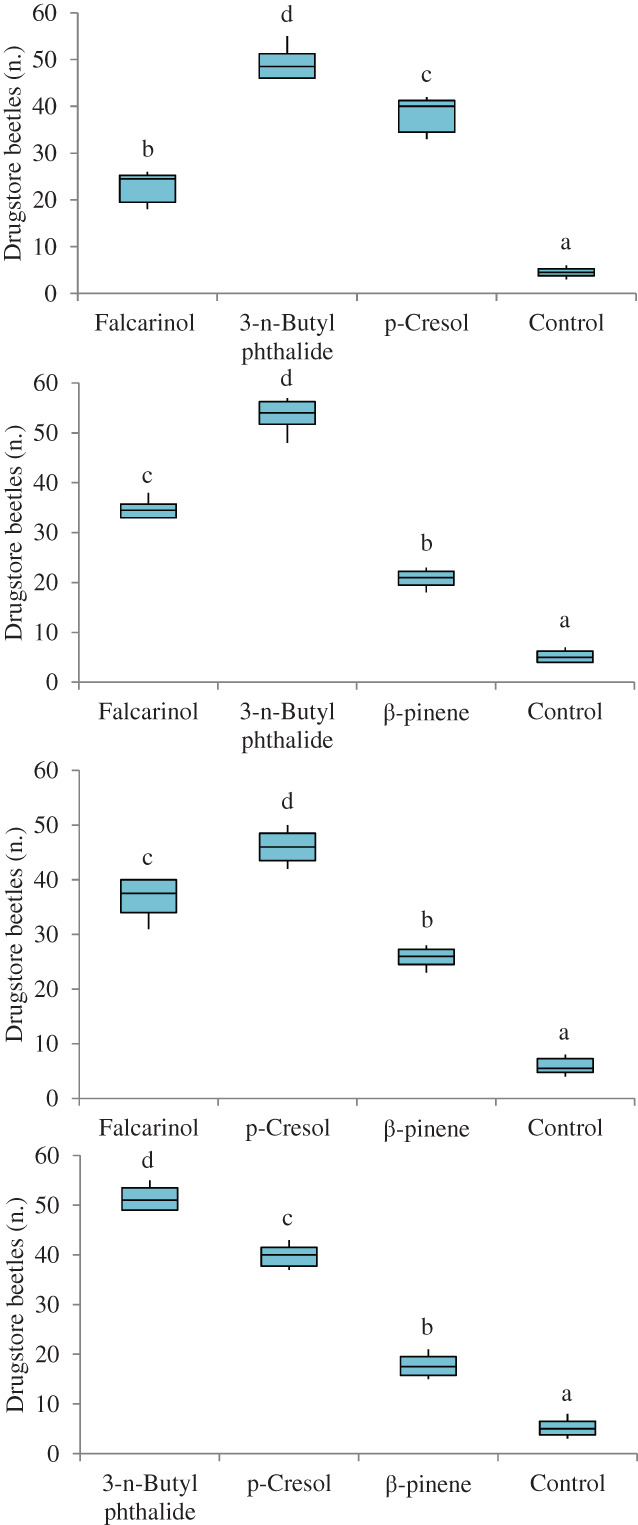
Olfactory responses of *S. paniceum* to falcarinol, 3‐*n*‐butyl phthalide, *p*‐cresol and β‐pinene (at their most attractive concentrations) in a four‐arm olfactometer. The control was mineral oil, and the tested concentrations for falcarinol, 3‐*n*‐butylphthalide, *p*‐cresol and β‐pinene were 50, 10, 50 and 100 μg μL^−1^, respectively. Each box plot represents the median and its range of dispersion (lower and upper quartiles and outliers). Above each box plot, different letters indicate significant differences (Wilcoxon test, *P* < 0.05).

Based on the results of six‐arm olfactometer bioassays, the most attractive doses of falcarinol, 3‐*n*‐butylphthalide, *p*‐cresol and β‐pinene were 500, 100, 500 and 1000 μg, respectively. Using these doses, the compounds were compared in multiple‐choice tests carried out in four‐arm olfactometer bioassays (Fig. 4). In all experiments, mineral oil, the control, was the least attractive stimulus chosen. The beetles significantly preferred 3‐*n*‐butylphthalide to *p*‐cresol and falcarinol (𝜒^2^ = 18.000, df = 3, *P* < 0.01), to falcarinol and β‐pinene (𝜒^2^ = 18.000, df = 3, *P* < 0.01), and to *p*‐cresol and β‐pinene (𝜒^2^ = 18.000, df = 3, *P* < 0.01). Insects were significantly more attracted to *p*‐cresol than to falcarinol and β‐pinene (𝜒^2^ = 18.000, df = 3, *P* < 0.01) (Fig. 4).

## 4 DISCUSSION AND CONCLUSIONS

Phytophagous insects rely on semiochemicals to locate suitable food materials, and mating and oviposition sites.^23^, ^24^, ^25^ Qualitatively and quantitatively different blends of volatiles from different plant species allow insects to discriminate and locate their preferred host plants.^25^, ^26^ The stored‐product pests, *Sitophilus oryzae* (L.),^27^ *S. zeamais* Motschulsky,^28^, ^29^ *S. granarius* (L.) (Colepotera, Curculionidae),^24^ *Oryzaephilus surinamensis* (L.) (Coleoptera, Silvanidae)^30^ and *Callosobruchus maculatus* (F.) (Coleoptera, Bruchidae),^31^ have been widely reported to be attracted to particular volatiles from some cereals and other stored products.

In our previous investigation, *S. paniceum* showed significant olfactory preferences for the volatiles of four CMPMs (i.e. *P. notoginseng*, *A. sinensis*, *G. elata* and *P. praeruptorum*). In the present study, falcarinol, 3‐*n*‐butylphthalide, *p*‐cresol and β‐pinene, as the most abundant volatiles of the aforementioned CMPMs, respectively, were shown to be perceived by the peripheral olfactory systems of *S. paniceum* males and females in a dose‐dependent manner.

Once the electrophysiological activity of the four compounds was ascertained, their biological activity was further investigated in six‐ and four‐arm olfactometer bioassays. All compounds were significantly attractive to *S. paniceum* in a range of concentrations (0.1–100 μg μL^−1^) and when the four compounds were compared at their optimally attractive concentrations, the order of olfactory preferences was 3‐*n*‐butylphthalide > *p*‐cresol > falcarinol > β‐pinene. Therefore, it seems that, amongst these volatiles, 3‐*n*‐butylphthalide has the greatest potential for development as a lure for *S. paniceum*. Although it is well‐known that the behavioural response elicited by a compound is not directly related to its electrophysiological activity, it is worth noting that, in the dose range tested, 3‐*n*‐buthylphthalide also was the strongest antennal stimulant of *S. paniceum* adults. This compound has a wide range of pharmacological effects and is widely used for the treatment of ischaemic stroke because of its low toxicity and safety.^32^ It also showed neuroprotection by reducing β‐amyloid‐induced toxicity in neuronal cells.^33^ Furthermore, it was shown to possess larvicidal and adulticidal activity against *Drosophila melanogaster* (Meig)^34^ and antifungal activity against *Candida albicans* (CP Robin) Berkhout.^35^ In a previous study, of 33 plant species tested, the *Angelica sinensis* essential oil (EO) showed the best repellent activity against *Aedes aegypti* (L.), and 3‐*n*‐buthylphthalide was identified as one of the main EO components.^36^ However, to the best of our knowledge, there are no reports investigating the behavioural response of any insect species to this compound.

More than 40 years ago, a female‐produced sex pheromone of *S. paniceum* was identified,^37^, ^38^ and has received commercial interest for pest monitoring in herbarium collections.^39^, ^40^, ^41^ Combined application of host plant volatiles and pheromones might be more effective for monitoring and control of *S. paniceum*, as shown for other pests.^27^, ^42^, ^43^, ^44^, ^45^, ^46^

Interestingly, the order of olfactory preference of *S. paniceum* for the odours of the above mentioned CMPMs previously was shown to be *P. notoginseng* > *A. sinensis* > *G. elata* > *P. praeruptorum*,^12^ whereas the preference ranking for their main components at the most attractive dose was 3‐*n*‐butylphthalide (*A. sinensis*) > *p*‐cresol (*G. elata*) > falcarinol (*P. notoginseng*) > β‐pinene (*P. praeruptorum*). This discordance might result from differences in the levels of the most abundant compounds among the volatiles from the four CMPMs, under natural conditions. In addition, although insects exhibited responses to individual compounds, their behaviour also can be influenced by specific blends of volatiles.^47^, ^48^, ^49^ Further research is needed to determine the behavioural activity of additional individual volatiles of these four CMPMs and their various blends to confirm the functional compounds and/or mixtures, which mediate the behavioural responses involved in the olfactory preferences of *S. paniceum* to different CMPMs. In addition, additive or synergistic attractants might be identified for the management of this pest beetle based on these bioassays.

This study confirmed that semiochemical volatiles participate directly in the interactions between *S. paniceum* and host CMPMs. Two parasitic wasps, *Lariophagus distinguendus* Forster and *Theocolax elegans* Westwood, which are natural enemies of *S. paniceum*, are attracted to volatiles of stored cereal grains which represent the preferred substrates of their hosts.^16^, ^22^, ^50^ Therefore volatile compounds identified from CMPMs that could be attractive to both *S. paniceum* and *S. paniceum* parasitoids deserve further research. This information could provide a theoretical framework for establishing a biocontrol system, based on CMPM volatiles, in which parasitoids are used to control *S. paniceum* infesting CMPMs.

Overall, this study demonstrated that semiochemical volatiles of CMPMs are involved in host‐plant selection by *S. paniceum*. It also showed how individual compounds previously identified as the main volatile components of different attractive CMPMs are able to stimulate the peripheral olfactory systems and elicit a positive chemotactic response in adult drugstore beetle. The most attractive 3‐*n*‐butylphthalide found in this study provides a basis for further field‐trapping experiments to develop semiochemically‐based monitoring tools and direct control options for *S. paniceum*.

## AUTHOR CONTRIBUTIONS

YC, GSG and CL conceived and designed the research; YBL, OMP, JW, ID and QQH conducted the experiments; YC and GSG analyzed the data; and YC, FM and GSG wrote the manuscript. All authors have read and agreed to the published version of the manuscript.

## CONFLICTS OF INTEREST

The authors declare no conflict of interest.

## Data Availability

The data that support the findings of this study are openly available in [repository name] at [DOI], reference number [reference number].
